# CD8^+^ T‐cell senescence and skewed lymphocyte subsets in young Dyskeratosis Congenita patients with *PARN* and *DKC1* mutations

**DOI:** 10.1002/jcla.23375

**Published:** 2020-05-25

**Authors:** Ting Zeng, Ge Lv, Xuemei Chen, Lu Yang, Lina Zhou, Ying Dou, Xuemei Tang, Jun Yang, Yunfei An, Xiaodong Zhao

**Affiliations:** ^1^ Pediatric Research Institute Ministry of Education Key Laboratory of Child Development and Disorders National Clinical Research Center for Child Health and Disorders (Chongqing) China International Science and Technology Cooperation base of Child Development and Critical Disorders Children's Hospital of Chongqing Medical University Chongqing China; ^2^ Chongqing Key Laboratory of Child Infection and Immunity Children's Hospital of Chongqing Medical University Chongqing China; ^3^ Department of Hematology and Oncology Children's Hospital of Chongqing Medical University Chongqing China; ^4^ Department of Rheumatology and Immunology Children's Hospital of Chongqing Medical University Chongqing China; ^5^ Department of Rheumatology and Immunology Shenzhen Children's Hospital Shenzhen China

**Keywords:** *DKC1*, Dyskeratosis Congenita, *PARN*, primary immunodeficiency, senescence, telomere

## Abstract

**Background:**

Dyskeratosis congenita (DC) is a syndrome resulting from defective telomere maintenance. Immunodeficiency associated with DC can cause significant morbidity and lead to premature mortality, but the immunological characteristics and molecular hallmark of DC patients, especially young patients, have not been described in detail.

**Methods:**

We summarize the clinical data of two juvenile patients with DC. Gene mutations were identified by whole‐exome and direct sequencing. Swiss‐PdbViewer was used to predict the pathogenicity of identified mutations. The relative telomere length was determined by QPCR, and a comprehensive analysis of lymphocyte subsets and CD57 expression was performed by flow cytometry.

**Results:**

Both patients showed typical features of DC without severe infection. In addition, patient 1 (P1) was diagnosed with Hoyeraal‐Hreidarsson syndrome due to cerebellar hypoplasia. Gene sequencing showed P1 had a compound heterozygous mutation (c.204G > T and c.178‐245del) in *PARN* and P2 had a novel hemizygous mutation in *DKC1* (c.1051A > G). Lymphocyte subset analysis showed B and NK cytopenia, an inverted CD4:CD8 ratio, and decreased naïve CD4 and CD8 cells. A significant increase in CD21^low^ B cells and skewed numbers of helper T cells (Th), regulatory T cells (Treg), follicular regulatory T cells (Tfr), and follicular helper T cells (Tfh) were also detected. Short telomere lengths, increased CD57 expression, and an expansion of CD8 effector memory T cells re‐expressing CD45RA (TEMRA) were also found in both patients.

**Conclusion:**

Unique immunologic abnormalities, CD8 T‐cell senescence, and shortened telomere together as a hallmark occur in young DC patients before progression to severe disease.

## 1 BACKGROUND

Dyskeratosis congenita (DC), a telomere disorder, is characterized by the classic triad of dysplastic nails, abnormal skin pigmentation, and oral leukoplakia.^1^ It was first described by Zinsser in 1906. Subsequent detailed confirmation by Engman in 1926^2^ and Cole in 1930^3^ led to its designation as Zinsser‐Engman‐Cole syndrome. Patients with DC with extremely short telomeres (<1st percentile according to age) are at an increased risk of bone marrow failure (pancytopenia), pulmonary fibrosis, malignancy, and other medical problems. Hoyeraal‐Hreidarsson syndrome (HHS) is a clinically severe variant of DC that typically presents early in childhood with cerebellar hypoplasia,^4^, ^5^ immunodeficiency, progressive bone marrow failure, and intrauterine growth retardation (IUGR).^6^ In recent years, pathogenic germline mutations associated with DC and related disorders have been identified in at least 14 different telomere biology genes.^7^, ^8^, ^9^, ^10^ Mutations in *DKC1* occur with a relatively high frequency in patients with the classical DC phenotype. Pathogenic variants of *PARN* are also known to cause telomere shortening and result in DC. Autosomal recessive inheritance of *PARN* variants was reported in 2015 and is extremely rare, with less than ten cases reported. Autosomal dominant inheritance of pathogenic variants of *PARN* can present as pulmonary fibrosis and/or bone marrow failure. Thirty percent of all DC patients continue to be genetically uncharacterized.^11^

Immunodeficiency is a major clinical feature of DC that can lead to significant morbidity and premature mortality. Lymphocytes are highly proliferative and are therefore particularly vulnerable to a decrease in telomerase activity. B and NK cells have been reported to be decreased in DC patients.^12^, ^13^ However, no detailed immunophenotyping has been performed in patients with DC.

## 2 MATERIALS AND METHODS

### 2.1 Human subjects

Both patients were under treatment at the Children's Hospital of Chongqing Medical University. Clinical data were collected during patient visits. In addition, healthy controls were recruited. All research studies were approved by the Medical Ethics Committee of the Children's Hospital of Chongqing Medical University.

### 2.2 Telomere length assessment and mutation analysis

Genomic DNA was isolated from whole blood using the QIAamp DNA mini kit (Qiagen Inc) according to the manufacturer's instructions. Total RNA was isolated from peripheral blood mononuclear cells (PBMCs) using the AxyPrep blood total RNA miniprep kit (Axygen Biosciences), and cDNA was synthesized using the EvoScript Universal cDNA Master (Roche). The relative telomere length (RTL) was measured from DNA from PBMCs using quantitative multiplex real‐time polymerase chain reaction (QPCR), as previously described.^14^ In‐house reference values were obtained from PBMCs of age‐matched healthy donors. The RTL was calculated as the median from at least three independent runs. Whole‐exome sequencing (WES) and direct sequencing were used to detect mutant sites, and the resulting protein structures were analyzed with Swiss‐Model and Swiss‐PdbViewer^15^, ^16^ based on the crystal structure of the protein database molecule 2a1s for *PARN* and 3uai for *DKC1*. Phylogenetic conversation was assessed using Bioedit.

### 2.3 Flow cytometry analysis of lymphocyte subsets

#### 2.3.1 Immunophenotyping

Lymphocyte subsets were analyzed as previously described.^17^ After red blood cell lysis, cells were detected on a FACSCanto II flow cytometer (BD Biosciences). T cells (CD3^+^) and the following T‐cell subsets were examined: naïve differentiated helper T lymphocytes (CD4 naïve; CD3^+^CD4^+^CD45RA^+^CD27^+^), central memory helper T lymphocytes (CD4 CM; CD3^+^CD4^+^CD45RA^−^CD27^+^), effector memory helper T lymphocytes (CD4 EM; CD3^+^CD4^+^CD45RA^−^CD27^−^), terminally differentiated effector memory helper T lymphocytes (CD4 TEMRA; CD3^+^CD4^+^CD45RA^+^CD27^−^), naïve differentiated cytotoxic T lymphocytes (CD8 naïve; CD3^+^CD8^+^CD45RA^+^CD27^+^), central memory cytotoxic T lymphocytes (CD8 CM; CD3^+^CD8^+^CD45RA^−^CD27^+^), effector memory cytotoxic T lymphocytes (CD8 EM; CD3^+^CD8^+^CD45RA^−^CD27^−^), terminally differentiated effector memory cytotoxic T lymphocytes (CD8 TEMRA; CD3^+^CD8^+^CD45RA^+^CD27^−^), TCRγδ^‐^ T lymphocytes (CD3^+^TCRγδ^−^), and TCRαβ + double‐negative T lymphocytes (TCRαβ + DNT: CD3^+^CD4^‐^CD8^‐^TCRαβ^+^TCRγδ^−^). B cells (CD19^+^) and the following B‐cell subsets were detected: switched memory B cells (CD19^+^CD27^+^IgD^−^), naïve B cells (CD19^+^CD27^−^IgD^+^), transitional B cells (CD19^+^CD38^+^CD24^+^), and plasmablasts (CD19^+^CD38^+^CD24^−^).

#### 2.3.2 B‐cell subsets

The analysis of B‐cell subsets was performed as described elsewhere,^18^, ^19^ with slight modifications. Isolated PBMCs were incubated with anti‐human CD19 (PerCP‐Cy5.5), anti‐human CD21 (PE), anti‐human CD27 (PE‐Cy7), anti‐human CD38 (PB), anti‐human IgD (BV510), and anti‐human IgM (APC) for 30 minutes on ice. After washing, cells were detected on a FACSCanto II flow cytometer (BD Biosciences).

#### 2.3.3 CD4 + T‐cell subsets

Human regulatory T cells (Tregs), follicular helper T cells (Tfh), and follicular regulatory T cells (Tfr) were detected by flow cytometry as previously described.^20^, ^21^ For analysis of Treg, isolated PBMCs were incubated with anti‐human CD4 (PE‐Cy7) and anti‐human CD25 (BV421) antibodies for 30 minutes at 20°C, and then fixed and permeabilized using the eBioscience Intracellular Fixation and Permeabilization kit (Thermo Fisher Scientific). Cells were then incubated with anti‐human FoxP3 (PE), anti‐human CD152 (APC), and anti‐human Helios (PerCP‐Cy5.5) antibodies for 30 minutes at 20°C. The cells were then analyzed on a FACSCalibur flow cytometer. For analysis of Tfh, whole blood was stained with anti‐human CD3 (PerCP), anti‐human CD4 (PE‐Cy7), anti‐human CXCR5 (BV421), anti‐human CD45RO (APC), anti‐human programmed cell death‐1 (PD‐1; FITC), and anti‐human inducible T‐cell co‐stimulator (ICOS; PE). For analysis of Tfr, whole blood was stained with anti‐human CD3 (PerCP), anti‐human CD4 (PE‐Cy7), anti‐human CXCR5 (BV421), anti‐human CD45RA (APC), anti‐human CD127 (PE), and anti‐human CD25 (APC). For analysis of Th subsets, whole blood was stained with anti‐human CD3 (PerCP), anti‐human CD4 (PE‐Cy7), anti‐human CXCR5 (BV421), anti‐human CD45RA (APC), anti‐human CXCR3 (APC), and anti‐human CCR6 (PE). After red blood cell lysis, cells were washed and detected on a FACSCantoII flow cytometer, and data were analyzed using FlowJo software (Tree Star, Ashland, OR, USA).

### 2.4 Analysis of CD57 expression

The expression of CD57 in T cells was also analyzed by flow cytometry.^22^ Isolated PBMCs were incubated with anti‐human CD4 (PE‐Cy7), anti‐human CD8 (BV421), and anti‐human CD57 (FITC) for 30 minutes on ice. After washing, cells were detected on a FACSCanto II flow cytometer.

### 2.5 Statistical analysis

The data were shown as mean ± standard deviation (SD), and Student's *t* test was used to determine the statistical significance of telomere length. Three independent experiments were performed. *P* < .05 was statistically considered significant.

## 3 RESULTS

### 3.1 Clinical phenotype and history of DC patients

**FIGURE 1:**
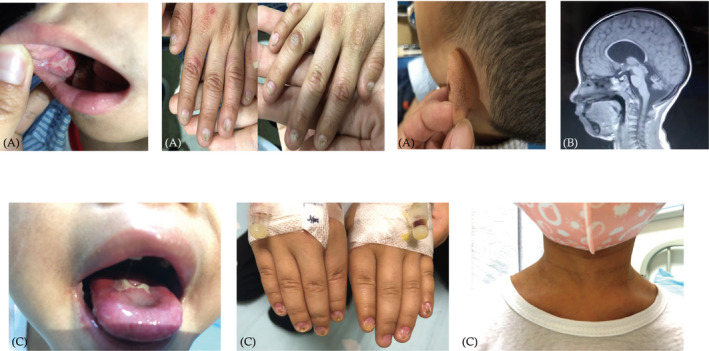
Clinical features of DC patients. A,B Oral lesions, dysplastic nails, abnormal skin pigmentation, and cerebellar hypoplasia (arrow) of P1. C, Oral leukoplakia, dysplastic nails, and abnormal skin pigmentation of P2

The clinical characteristics of the two patients evaluated in this study are summarized in Table 1. P1 was born at term to healthy non‐consanguineous parents from China and was delivered by Cesarean section at full term, weighing 2.5 kg and was vaccinated at birth with BCG without adverse effects. He initially developed oral lesions at the age of 2 years. Nail dystrophy and skin pigmentation were first documented at age 3 and 7, respectively (Figure 1A). At 5 years of age, the patient was found to have persistent thrombocytopenia (50 × 10^9^/L), high IgE titers (2500‐3500 IU/mL), B/NK cell lymphopenia, and transient Epstein‐Barr viremia without clinic manifestation. In addition, the manifestation of cerebellar dysplasia (Figure 1B), microcephaly, and growth retardation led to the clinical diagnosis of HHS.

**TABLE 1:** Clinical phenotype

	P1	P2
Age	7y10m	7y5m
Gender	M	M
Abnormal nails	^+^	^+^
Skin pigmentation	^+^	^+^
Oral leukoplakia	^+^	^+^
Microcephaly	^+^	^+^
Cerebellar hypoplasia	^+^	/
Developmental delay	^+^	^+^
Pulmonary fibrosis	−	−
Hematological diseases	PLT: 40‐60 × 10^9^/L↓	HB: 25g↓, PLT: 10‐30 × 10^9/^L↓
Severe enteropathy	−	−
Malignancy	−	−
Immunoglobulin	IgE↑, IgM↓	IgA↑

Abbreviations: HB, hemoglobin (N:120‐160g); PLT, blood platelet (N: 100‐300 × 10^9^/L).

P2 was born to non‐consanguineous parents with an uneventful family history and had a healthy older sister. Post‐natal development was normal and was vaccinated with no adverse effects. The patient was referred to us for pancytopenia with petechia at the age of 7 years. At the same time, the patient was found to have the classical triad of DC symptoms (Figure 1C), but the onset of the symptoms is unknown. Further investigation of bone marrow cytology indicated aplastic anemia. As in P1, microcephaly and growth retardation were also found. Moreover, the patient was diagnosed with a fungal infection of the skin. After treatment with erythrocyte and platelet infusion, the patient's erythrocyte and platelet count increased, but did not return to normal levels.

### 3.2 Severe telomere shortening and computational analysis of mutations

**FIGURE 2:**
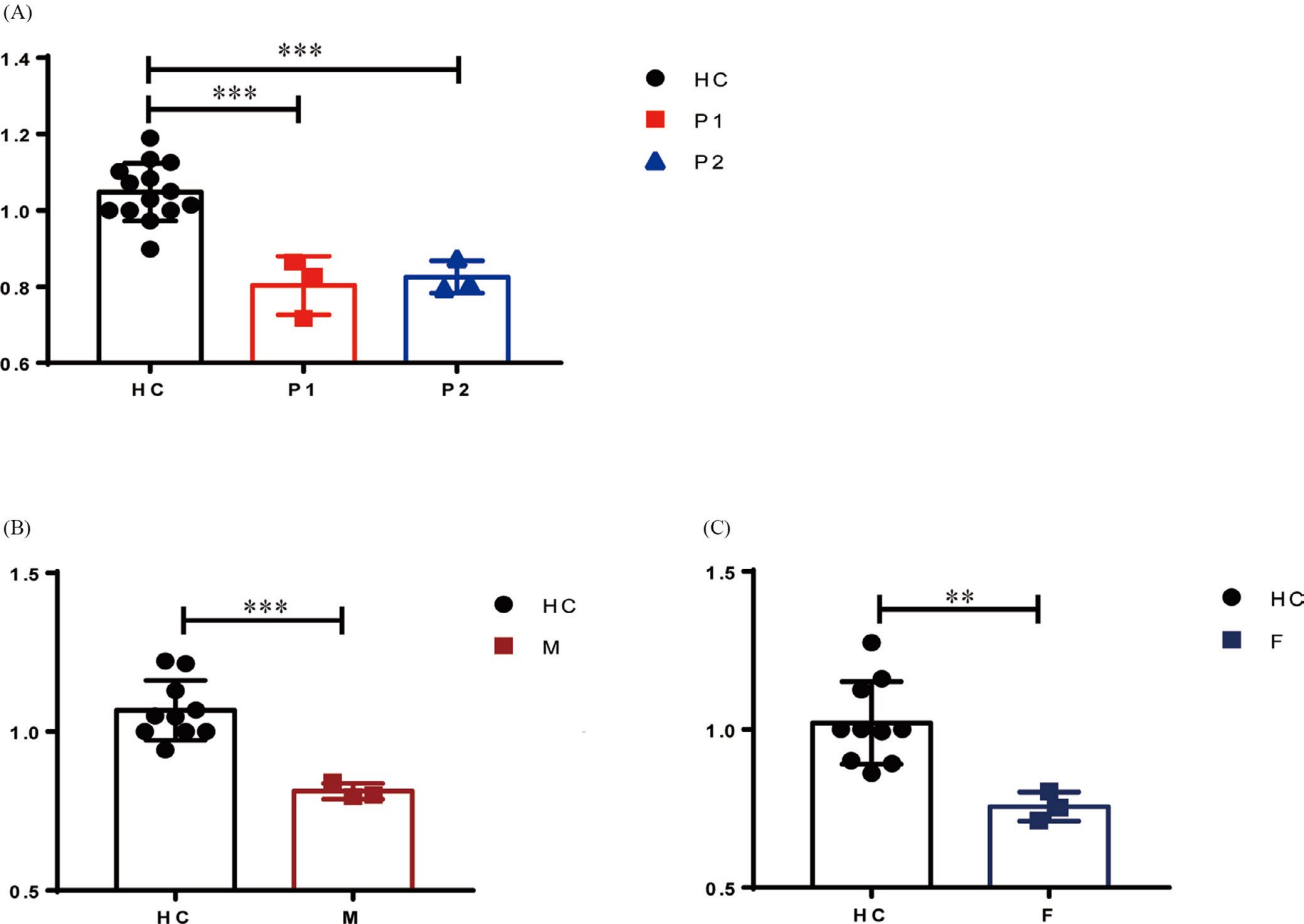
Relative telomere lengths (RTL). RTL were measured in the PBMCs of P1, P2 (A) and the parents of P1 (B, C) using qPCR, and were compared with age‐matched, healthy controls (HC). *P < .05, **P < .01, ***P < .001, by two‐tailed *t* test

**FIGURE 3:**
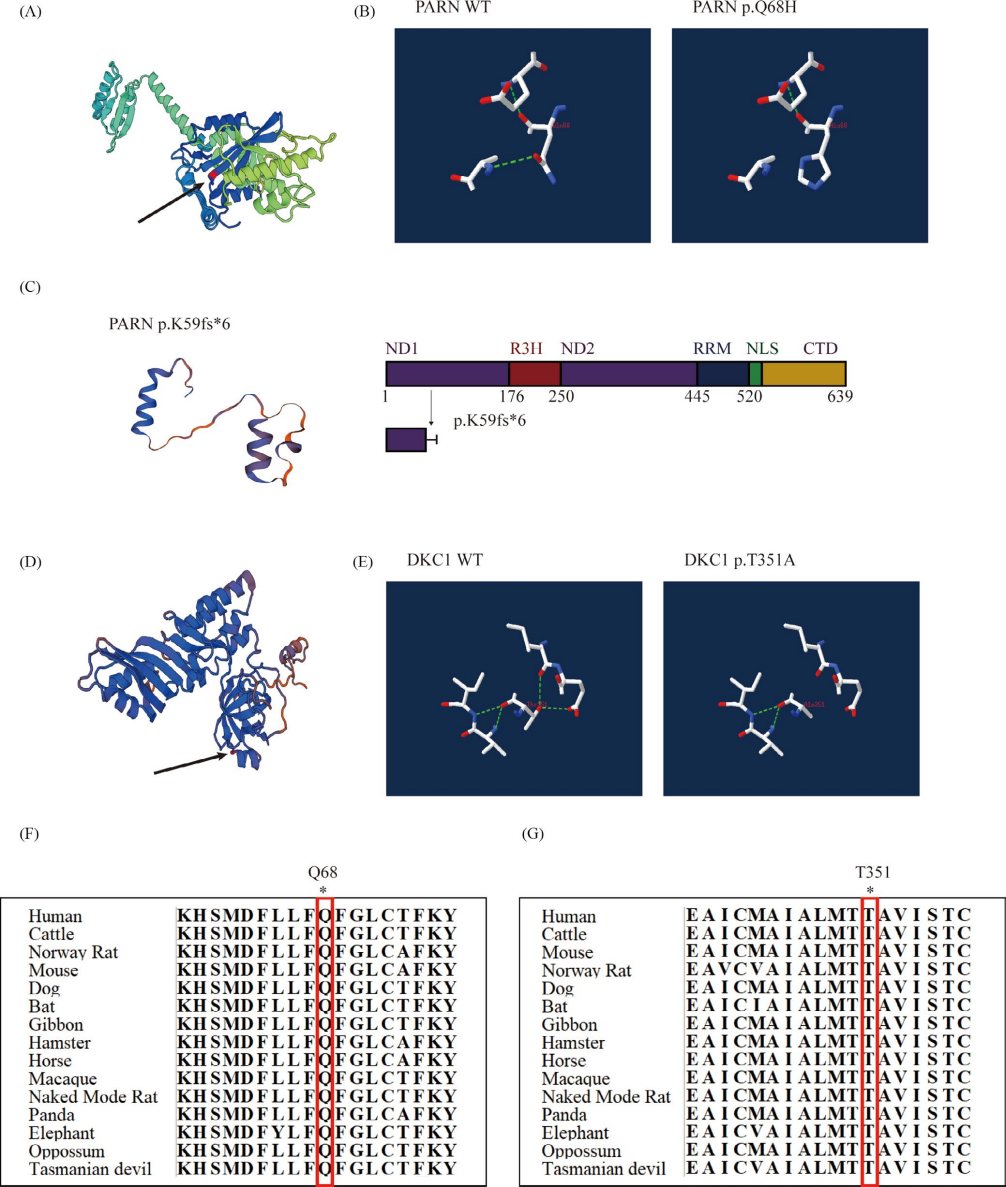
Structure prediction and evolutionary conservation of mutations. A, The mutated amino acid (p.Q68H) of *PARN* is predicted to be located in a β‐sheet of a β‐meander motif by Swiss‐Model based on template. B, The structural impact of the Q68H variant was analyzed using template 2a1s from Protein Databank (PDB). Residue 68 and nearby residues within associated H‐bonds were modeled in wild‐type (WT) and variant *PARN* using Swiss‐PbdViewer. C, Comparing with WT protein structure of *PARN* in (A), this deletion mutation causes a dramatical change. A linear diagram of the *PARN* protein shows only partial ND1 of *PARN* left. D, The mutated amino acid (p.T351A) of *DKC1* is predicted to be located between a β‐sheet and an α‐helix by Swiss‐Model. E, The structural impact of the T351A mutation was analyzed using template 3uai from PDB. The analytical method is the same as in (B). Computed hydrogen bonds are shown as green dashed lines. F, p.68Q and (G) p.351T are highly conserved across 14 species

**FIGURE 4:**
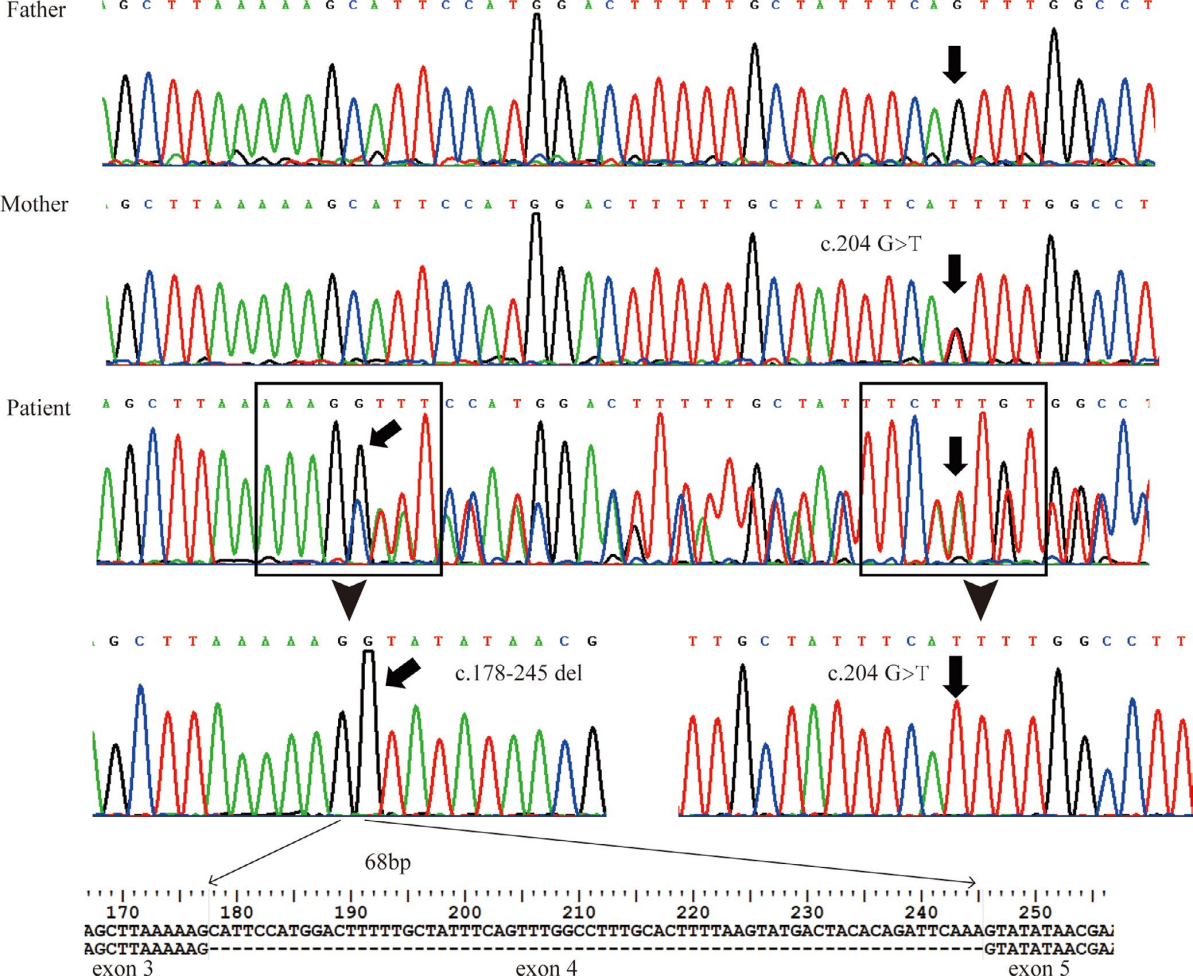
Sequence analysis of *PARN* in P1' s family. Compound heterozygous mutations of PARN was identified in P1. His mother was a carrier, and his father was a wild type

Both patients and the parents of P1 exhibited short telomeres (compared with age‐matched controls respectively), as measured by qPCR of PBMCs (*P* < .001 in patients and P1's mother, *P* < .01 in P1's father) (Figure 2). WES analysis revealed a heterozygous mutation in exon4 of *PARN* (NM_ 002582) c.204G > T (p.Q68H) in P1 inherited from his mother. This mutation is located in the β‐fold region of *PARN* (Figure 3A), predicted to lead to the loss of a hydrogen bond in the protein structure and is highly conserved in all 14 species (Figure 3B,F). The pathogenicity of the biallele mutation at this locus has been demonstrated in a Hoyeraal‐Hreidarsson patient reported by Benyelles.^23^ Considering P1 also showed the manifestations of Hoyeraal‐Hreidarsson syndrome, we performed direct sequencing of the full *PARN* coding sequence in P1 and his parents. After that, a deletion of exon 4 (c.178‐245del) was found in P1 (Figure 4), resulting in a frameshift deletion and a premature stop codon (p.K59fs*6), which cause only partial ND1(N‐terminal nuclease domains 2) of *PARN* left and is predicted to make a dramatical change in the protein structure(Figure 3C). In addition, P2 showed a variant, c.1051A > G (p.T351A), in exon 11 of the *DKC1* gene (NM_001363) by whole‐exome sequencing, which has not been reported before. The variant is located at the C‐terminal region of the helix structure of *DKC1* and is predicted to result in the loss of two hydrogen bonds (Figure 3D,E). This mutation affects the region with a very high degree of homology and conservation in all 14 species (Figure 3G).

### 3.3 Immunological findings

#### 3.3.1 Analysis of lymphocyte subsets

Both patients showed an increased frequency of T cells but a decreased frequency of B cells and NK cells, and a skewed ratio of CD4 to CD8 T cells. Among T cells, the frequency of CD8 cells in P1 was increased, mainly due to a dramatic increase in the frequency of TEMRA and CD8 CM, while the relative abundance of naïve CD8 T cells was decreased. The frequencies of total CD4 cells and naïve CD4 cells were decreased, but the proportions of CD4 CM, EM, and TEMRA among total CD4 T cells were all increased. Like P1, P2 showed an increase in the proportion of CD8 cells, mainly due to an increase in CD8 TEMRA, CM, and EM, and the proportion of naïve CD8 cells was also decreased. Although the proportion of CD4 TEMRA was increased, the frequency of total CD4 cells was normal. In addition, the proportion of γδT cells in P2 was also slightly increased. Furthermore, the absolute numbers of most cell subsets in P2 were decreased due to the pancytopenia. Among B cells, both patients showed the decrease in frequency of Naïve B cells while others have varied degrees of increase. These data are summarized in Table 2.

**TABLE 2:** Immunological phenotype

Lymphocyte subsets	%	%	% Reference	Absolute count (/μL)	Absolute count (/μL)	Reference (/μL)
	P1	P2		P1	P2	
T cells (/μL)	95.31↑	94.8↑	60.05‐74.08	2887.9↑	1649.5	1424‐2664
CD8 T cells (/μL)	70.7↑	59.7↑	19.68‐34.06	2142.2↑	1039.3	518‐1125
CD8 naïve (/μL)	18.8↓	27.1↓	41.58‐77.90	402.7	281.7↓	297‐730
CD8 TEMRA (/μL)	40.4↑	42.6↑	1.70‐24.62	865.5↑	442.7↑	11‐218
CD8 CM (/μL)	30.6↑	22.2	12.08‐30.54	655.5↑	230.7	85‐268
CD8 EM (/μL)	10.3	8.1	1.58‐13.18	220.6↑	83.8	10‐129
CD4 T cells (/μL)	16.99↓	28.0	26.17‐40.76	514.8↓	487.9↓	686‐1358
CD4 naïve (/μL)	30.1↓	56.7	45.56‐75.28	155↓	276.6↓	321‐972
CD4 TEMRA (/μL)	3.7↑	2.9↑	0.00‐1.06	19.1↑	14.2	0‐13
CD4 CM (/μL)	56.4↑	36.6	22.06‐46.46	290.3	178.6↓	211‐478
CD4 EM (/μL)	9.84↑	3.8	2.08‐8.78	50.7	18.6↓	23‐84
TCR αβ DNT (/μL)	0.6	0.9	0.18‐2.81	17.9	14.8	4‐55
TCR γδ T cells (/μL)	15.4	20.6↑	6.92‐19.84	444.7↑	339.8	124‐410
B cells (/μL)	2.1↓	2.7↓	10.21‐20.12	63.9↓	47.2↓	280‐623
Memory B cells (/μL)	53.7↑	36.1↑	7.76‐19.90	34.3	17.0↓	31‐94
Naïve B cells (/μL)	21↓	32.4↓	48.36‐75.84	13.4↓	15.3↓	147‐431
Transitional B cells (/μL)	20.3↑	3.8	2.58‐12.30	13.0	1.8↓	10‐66
Plasmablasts B cells (/μL)	38.4↑	34.2↑	0.90‐7.36	24.6	16.1	4‐28
NK cells (/μL)	2.42↓	2.5↓	9.00‐22.24	73.3↓	42.6↓	258‐727
CD4/CD8	0.24↓	0.47↓	0.87‐1.94			

Abbreviations: CM, central memory T lymphocyte; DNT, double‐negative T lymphocyte; EM, effector memory T lymphocyte;TEMRA, terminally differentiated effector memory T lymphocyte.

#### 3.3.2 Analysis of B‐cell subsets

**FIGURE 5:**
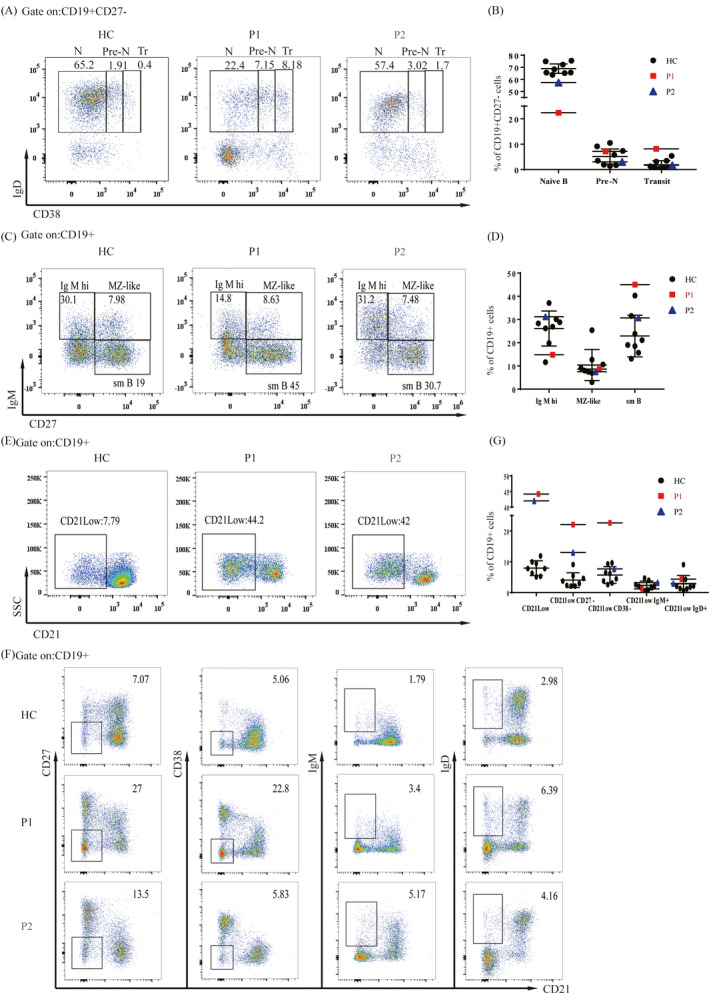
Characterization of B‐cell subsets. A, Peripheral blood CD19^+^CD27^‐^ B cells were gated into CD38^low^ (naïve, N), CD38^int^ (pre‐naïve, Pre‐N), and CD38^hi^ (transitional, Tr) subsets. B, Both P1 and P2 showed a decreased naïve cell population, and transitional B cells were increased in P1 compared with age‐matched controls. C, B cells were assessed for the distribution of IgM^hi^, marginal zone‐like (Mz‐like), and switch memory (sm) B cells after staining for the expression of CD19, CD27, and IgM. D, P1 showed an increase in the proportion of sm B cells and a slight decrease in IgM^hi^ cells, while P2 showed normal frequencies of these subsets. E‐F, Both P1 and P2 showed significantly increased percentages of CD21^‐^ B cells. Increased proportions of CD21^‐^CD27^‐^ B cells and CD21^‐^CD38^‐^ B cells were found in P1, while CD21^‐^CD27^‐^ B cells were increased in P2

We used the methods in the literature to further distinguish the subsets of B cells.^18^, ^19^ Naïve B cell numbers were decreased in both patients (Figure 5A,B). Moreover, more detailed clustering revealed that the proportions of switch memory (sm) B cells and transit B cells were increased in P1 (Figure 5C,D), and the proportion of CD21^low^ B cells was significantly increased in both patients (Figure 5E). Both CD21^‐^CD27^‐^ B cells and CD21^‐^CD38^‐^ B cells were increased in P1, while only CD21^‐^CD27^‐^ B cells were significantly increased in P2 (Figure 5F,g).

#### 3.3.3 Analysis of CD4 T‐cell subpopulations

**FIGURE 6:**
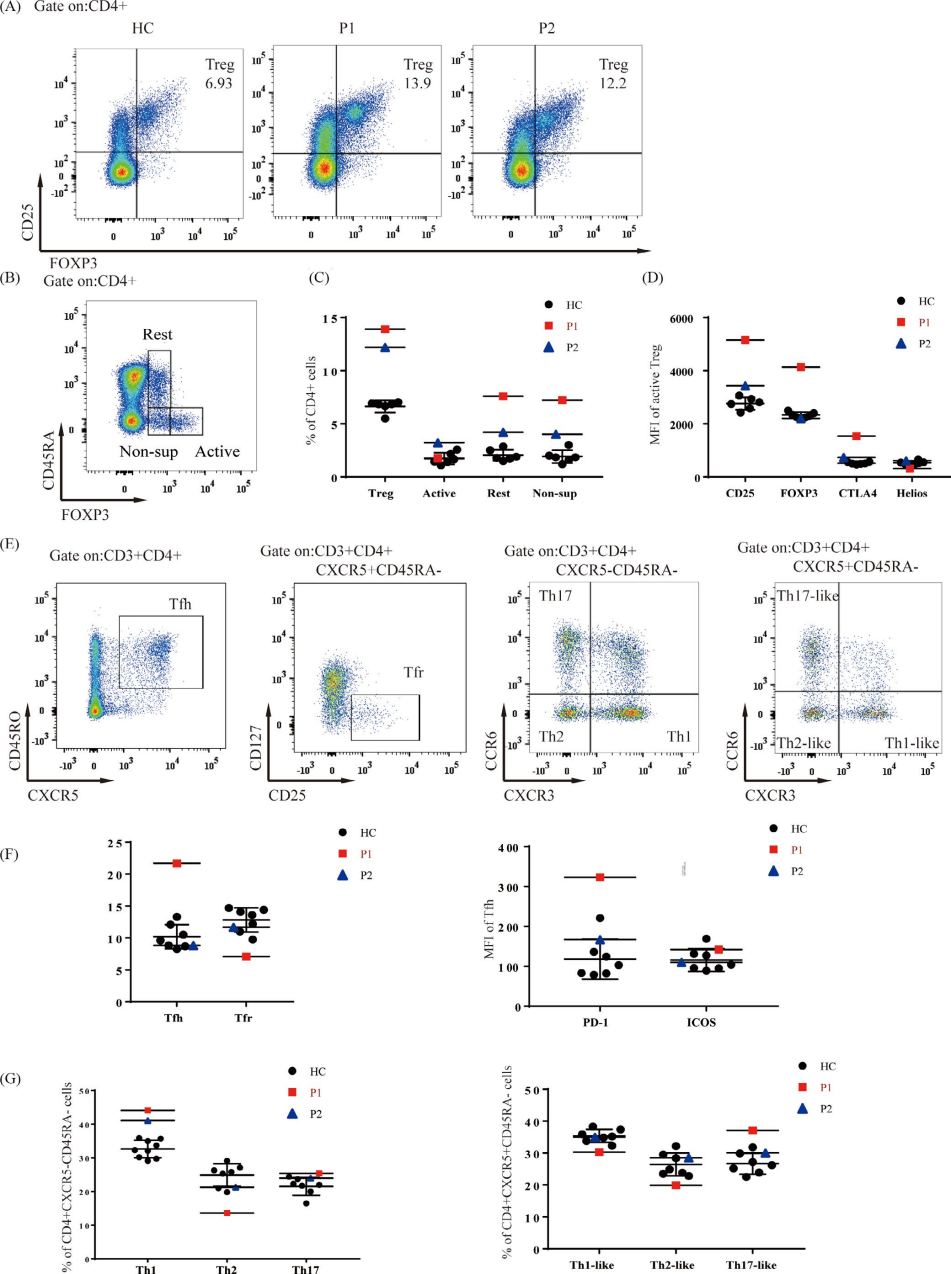
Characterization of CD4 T‐cell subsets. A, The frequencies of total Treg in P1 and P2 were increased compared with HC. B, Detailed phenotyping of Treg subsets by flow cytometry. C, The increase in total Treg was mainly due to an increase in the proportion of resting and non‐sup Treg. D, The expression of CD25, FoxP3, and CTLA‐4 was increased in activated Treg cells in P1, but only slightly elevated CD25 expression was found in P2. E, Detailed method for identifying Tfh, Tfr, Th1/2/17, and Th1/2/17‐like cells by flow cytometry. F, P1 showed an increased percentage of Tfh and increased Tfh expression of PD‐1. G, Frequencies of the indicated Th subsets in P1 and P2

The proportion of Treg was increased in both patients (Figure 6A), mainly due to an increase in resting and non‐sup Treg, when Treg was subdivided into CD45RA ^−^ Foxp3^+^ activated Treg (aTreg), CD4^+^CD45RA^+^Foxp3^+^ resting Treg (rTreg), and CD45RA−Foxp3− non‐suppressive Treg (Figure 6B,C). In addition, the expression of CD25, FoxP3, and cytotoxic T lymphocyte‐associated‐4 (CTLA‐4) was increased in active Treg cells from P1, but in P2, only the expression of CD25 was slightly increased (Figure 6D). In addition, we further analyzed the percentage of Tfh, Tfr, and their subgroups by using the gating method as shown in the Figure 6E and found in P1, but not P2, the percentage of Tfh was increased, and the percentage of Tfr was decreased (Figure 6E,F). The expression of PD‐1 on Tfh was increased in P1 only (Figure 6E,F). Furthermore, in the analysis of Th subpopulations, the proportions of Th1 and Th17‐like cells were increased and the proportions of Th2, Th1‐like, and Th2‐like cells were decreased in P1, while in P2, only the proportion of Th1 cells was increased (Figure 6E,G).

#### 3.3.4 Expression of CD57 in CD4 and CD8 T cells

**FIGURE 7:**
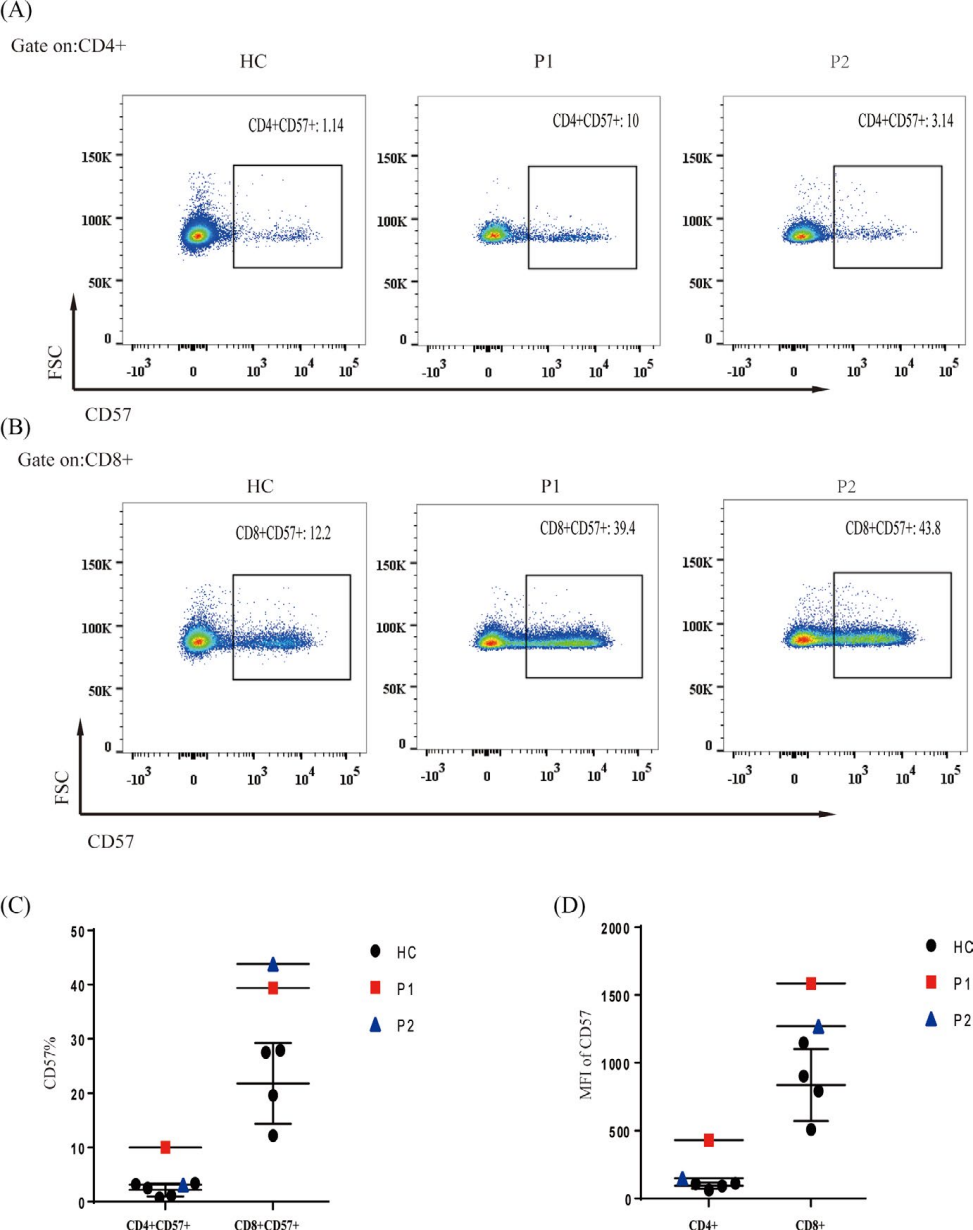
Expression of CD57. A‐D, Compared with healthy controls, P1 showed increased expression of CD57 in CD4^+^ and CD8^+^ T cells, while P2 only showed an increase in CD57^+^ CD8^+^ T cells

The expression of CD57 was increased both in CD4 and CD8 T cells in P1, but in P2, it was only increased in CD8 T cells (Figure 7A‐C). Analysis of the percentage of CD57^+^ cells as well as the mean fluorescence index (MFI) for CD57 expression showed a similar trend (Figure 7D).

## 4 DISCUSSION

Dyskeratosis congenita is a rare inherited bone marrow failure syndrome with multi‐system involvement, caused by premature attrition of the protective telomeres at the ends of linear chromosomes. The clinical diagnosis of DC requires the presence of at least two of the three classic mucocutaneous features and two or more somatic abnormalities.^10^, ^11^, ^12^ HHS is a severe form of DC characterized by IUGR, microcephaly, cerebellar hypoplasia, moderate to severe mental retardation, progressive immunodeficiency, and pancytopenia. In our study, both patients showed thrombocytopenia as well as the classic triad of DC (Figure 1). In addition, P2 had decreased white blood cell counts and hemoglobin levels with bone marrow cytology indicating aplastic anemia. Moreover, both patients showed microcephaly and growth retardation. P1 suffered typical cerebellar atrophy and was clinically diagnosed with HHS. Neither patient has experienced a life‐threatening infection so far and both them were normal with vaccine. Dysgammaglobulinemia (low IgG, IgM, or IgA) was a common finding in previous studies.^12^ However, P1 showed a persistent increase in IgE and a decrease in IgM, while P2 showed an increase in IgA (Table 1), although both patients had decreased peripheral B cell counts. These observations demonstrate the clinic diversity of DC and may indicate age or race‐related characteristics.

*PARN* mutations are extremely rare and the bilallelic pathogenic variants in *PARN* often been found in HHS patient. *PARN* protein is mainly made up by 6 domains, including N‐terminal nuclease domains (ND1 and ND2), conserved arginine and 3‐histidine containing domain(R3H), RNA recognition motif (RRM), nuclear localization signal (NLS), and C‐terminal domain (CTD).^24^ In our study, consistent with previous reports, a compound heterozygous mutation was eventually confirmed in P1 with HHS characteristics, including one site mutation (c.204G > T, p.Q68H) that has been reported to be pathogenic and the other deletion mutation (c.178‐245del, p.K59fs*6) causing only partial ND1(N‐terminal nuclease domains 2) of *PARN* left and a dramatical change in the protein structure. At the same time, his mother, a carrier of monoallelic *PARN* mutation, showed no obvious clinical phenotype with short telomere, which is consistent with the conclusion of incomplete penetrance of *PARN*‐associated disease in previous study.^25^ Mutations in *DKC1* were the first disease‐causing mutations identified and are associated with the classic DC phenotype. The *DKC1* mutations identified in P2 with a very high degree of homology and conservation in all 14 species were predicted to lead to loss of a hydrogen bond, resulting in significant changes to the secondary structure of the protein.

Although immunodeficiency is the main cause of premature death in patients with DC, immunological variation has not attracted enough attention in the clinical diagnosis and treatment of DC patients.^26^ The immunologic features of the unique T^+^B^low^NK^low^ immune phenotype were reported recently by Jyonouchi el al and Touzot et al^12^, ^27^ and were found in both of our patients supporting the diagnosis of DC. We performed a detailed immunophenotype analysis of two young DC patients and found a decrease in naïve T cells and an increase in effector and central memory T cells in both patients, which may lead to an increased risk of opportunistic infection in children, but the patients in our study have not yet developed a fatal infection. A recent study showed that shortened telomeres lead to decreased thymic output, resulting in a depletion of naïve CD4 and CD8 T cells and an increase in CD8 TEMRA.^28^, ^29^ This is consistent with our findings. Both patients showed a dramatic increase in CD21^low^ cells in addition to a striking reduction in naïve B cells. Further analysis of this population showed an increase in CD21^low^CD27^‐^, and CD21^low^CD38^‐^ cells in P1, while P2 only showed a slight increase in CD21^low^CD27^‐^ cells. P1 also showed a decrease in IgM^hi^ and sm B cells. In addition, P1 had elevated levels of serum IgE and decreased levels of serum IgM, while IgA levels were elevated in P2, indicating aberrant humoral immunity in young DC patients. CD21^low^ B cells are elevated in many patients with autoimmune disease.^19^, ^30^, ^31^ Although our patients showed no significant signs of autoimmune disease, aplastic anemia, bone marrow failure, and pulmonary fibrosis occur frequently in patients with immune dysregulation. We therefore also analyzed the frequency of Tfh and Treg subsets in these patients, which are closely related to the regulation of immune function.^20^, ^21^, ^32^ P1 showed significant increases in Tfh numbers and PD‐1 expression, and a decrease in Tfr numbers, as well as increased numbers of Th1 and Th17‐like cells and decreased numbers of Th2, Th1‐like, and Th2‐like cells. By contrast, P2 only showed increases in Th1 cells. Both patients had elevated Treg numbers. In P1, the numbers of non‐sup and resting Treg were increased, and the expression of functional molecules on activated Treg was increased, while all Treg subtypes were increased in P2. This may suggest that telomere shortening leads to excessive or aberrant activation of the immune system, causing a compensatory increase in Treg, which inhibits the activation of other immune subsets, preventing obvious symptoms of immune dysfunction. However, as the disease progresses, and T/B‐cell output decreases but T‐cell senescence increases, immune dysregulation occurs, resulting in the severe symptoms of DC.

It is known that with every cell division, 50‐200 bases of DNA are lost due to incomplete terminal replication of the daughter strand. As cell division continues, the telomeres shorten, and once a critical length is reached, cell senescence/apoptosis will be triggered.^33^ DC is caused by germline mutations in genes associated with regulation of telomere length, resulting in very short telomeres. Both patients in our study had short telomeres, consistent with previous reports.^11^, ^24^, ^34^ Because the telomere length is highly variable according to race, age, and disease state, the cell surface marker CD57 is routinely used to identify terminally differentiated, senescent cells with reduced proliferative capacity and altered functional properties.^22^ The expression of CD57 was dramatically elevated in CD4 and CD8 T cells in both patients in our study. Given the reduced telomere lengths, the increased expression of CD57 on CD8 T cells, and the increased frequency of CD8 TEMRA, our findings suggest that CD8 T‐cell senescence may be another hallmark of DC.

In summary, we provide a thorough analysis of the clinical symptoms, genetic mutations, and immunophenotype in two cases of typical DC. Our study provides additional information on immune dysfunction and CD8 T senescence may be hallmark of DC in young patients without severe immunodeficiency or definite molecular diagnosis, especially 30% of DC patients reported were without of known pathogenic genes. Given the extremely low disease incidence of DC, more case studies are needed to draw more solid conclusions.

## AUTHOR CONTRIBUTIONS

We thank Xiaodong Zhao for guiding the study and providing the financial supporting. Xiaodong Zhao and Yunfei An designed the study. Ting Zeng, Ge Lv, Xuemei Chen, Lu Yang performed experiments. Ting Zeng collected and analyzed the data. Lina Zhou provided the technical guidance of Flow cytometry. Ting Zeng wrote the manuscript, and Xiaodong Zhao, Yunfei An, Xuemei Tang, Ying Dou, and Jun Yang checked and revised the manuscript.
